# Towards Open and Equitable Access to Research and Knowledge for Development

**DOI:** 10.1371/journal.pmed.1001016

**Published:** 2011-03-29

**Authors:** Leslie Chan, Barbara Kirsop, Subbiah Arunachalam

## Abstract

Leslie Chan and colleagues discuss the value of open access not just for access to health information, but also for transforming structural inequity in current academic reward systems and for valuing scholarship from the South.

**Summary Points**

- Unequal access to and distribution of public knowledge is governed by Northern standards and is increasingly inappropriate in the age of the networked “Invisible College”.
- Academic journals remain the primary distribution mechanism for research findings, but commercial journals are largely unaffordable for developing countries; local journals—more relevant to resolving problems in the South—are near-invisible and under-valued.
- Donor solutions are unsustainable, are governed by markets rather than user needs, and instil dependency.
- Open access is sustainable and research driven and builds independence and the capacity to establish a strong research base; it is already converting local journals to international journals.
- However, as open access becomes the norm, standards for the assessment of journal quality and relevance remain based on Northern values that ignore development needs and marginalise local scholarship.

There is growing recognition that the capacity to conduct research and to share the resulting knowledge is fundamental to all aspects of human development, from improving health care delivery to increasing food security, and from enhancing education to stronger evidence-based policymaking. Today, the primary vehicle for disseminating research is still the peer-reviewed journal, which has retained much of its traditional form and function, although now it is largely digital. But despite improved access to the Internet, researchers in the developing world continue to face two problems—gaining access to academic publications due to the high cost of subscriptions, and getting their research published in “international” journals, because their work is either considered to be only of local or regional interest or does not meet the quality standards required by the major commercial indexes. The cartographic representation of the world according to the volume of publications from each country in early 2000 starkly depicts a world of highly unequal contribution and participation in science (Figure 1).

**Figure 1:**
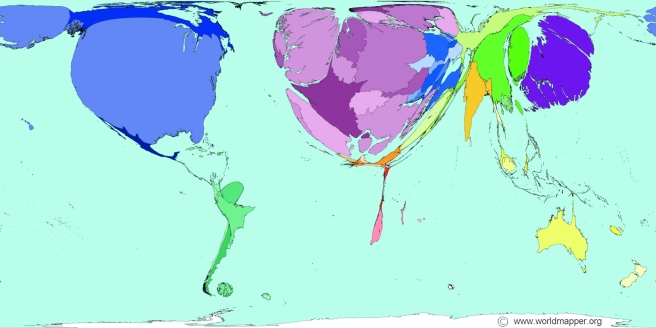
Unequal contribution and participation in science. Image © Copyright SASI Group (University of Sheffield) and Mark Newman (University of Michigan). Available: http://www.worldmapper.org/display.php?selected=205. The authors have been granted permission to reproduce this figure under the terms of the Creative Commons Attribution License, which permits unrestricted use, distribution, and reproduction in any medium, provided the original author and source are credited. Source of data used to create map: World Bank's 2005 World Development Indicators.

This inequity has led to the misguided notion that little, if any, research of substance is generated in the global South, and that the needs of researchers in poor countries are therefore met solely by information donation from the North. The one-way North to South flow of knowledge is not all that is necessary for development, and the Research4Life program only addresses part of the problem (http://www.research4life.org/). The Research4Life program is the collective name for three journal access programs–HINARI, AGORA, and OARE—and comprises a public–private partnership between major commercial publishers and three United Nations (UN) agencies (Box 1).

**Box 1. Main Journal Access Programs**

- HINARI (Health InterNetwork Access to Research Initiative) is managed by the World Health Organization [WHO] in partnership with Yale University Library and the program provides free or very low cost online access to the major journals in biomedical and related social sciences to local, not-for-profit institutions in qualified low income countries.
- AGORA (Access to Global Online Research in Agriculture) is managed by the Food and Agriculture Organization in partnership with Cornell University and provides access to over 1,200 international journals covering agriculture, fisheries, food, nutrition, veterinary science, and related biological, environmental, and social sciences. It also includes several important databases and indexes.
- OARE (Online Access to Research in the Environment) is coordinated by the United Nations Environment Programme at Yale University and provides access to more than 2,000 scientific journals in a wide range of disciplines contributing to our understanding of the natural environment.

The recent announcement by the commercial publisher Elsevier (a HINARI founding partner) of withdrawal of access to their journals from Bangladeshi institutions, and the subsequent announcement that Bangladesh is in transition towards a paid licensing scheme [1], is sobering. It reminds us that large multinational publishers are driven primarily by commercial motives and market shares, and that HINARI may be serving as a marketing device to prepare the ground for national site licenses in the countries with rising GDP or growing research needs. Site licensing is a standard subscription practice of commercial publishers for providing institution-wide electronic access to their journals. Fees for site licensing generally vary according to the number of institutional users. It is also common for large multinational publishers to combine all of their journal holdings into one large “take-it-or-leave-it” bundle, often referred to as the “Big Deal” [2]. While the Big Deal is a legitimate commercial strategy, even rich institutions in the North can ill-afford the continuing rising cost. It is very clear that for low-income countries, the so-called information philanthropy [3] is not a long-term sustainable solution to ensure access to publicly funded research publications, a prerequisite for developing a strong and independent research base.

## Misguided Dependencies on Free Subscriptions

Coming as these programs do with the blessings of the UN agencies and powerful commercial publishers, it has been hard to wean research communities off dependency systems and onto true open access (OA) resources. These resources include the growing number of OA journals and institutional repositories worldwide that are now accessible free of cost to anyone with Internet access. The growing volume of OA resources provides a far greater degree of freedom for researchers to exchange and collaborate, for knowledge to be translated into useable forms by frontline health workers, and for emerging technologies such as text mining and semantic tagging for faster knowledge discovery to be used. It must be underscored that such usages and redistribution are not permitted by donated content included in the Research4Life programs, even though users are free to read such content. Further, while the ”free access” programs purport to be providing essential articles to researchers in poor nations (excluding countries such as India where the publishers have an existing market), access is not country-wide, but is only available if the researchers work in the registered institutions.

## South–South Collaborations

For scholarly publishers and researchers in the South, OA is particularly important because it provides an unprecedented opportunity for South–South exchange and for local research to become an integral part of the global knowledge commons. More importantly, research findings from regions with similar socioeconomic conditions may be far more relevant than research from the richer countries. This is particularly true with health care and medical treatments.

Take, for example, the journal *African Health Sciences*, edited by Dr. James K. Tumwine and published by the Faculty of Medicine at Makerere University in Uganda. This 10-year-old journal is thriving on the Web (http://www.bioline.org.br/hs) and gaining international recognition and global usage, showing that OA is not only viable, but with time will become the norm. The journal is one of a small number of African-based journals indexed by Medline, and the journal content is also archived in PubMedCentral (http://www.ncbi.nlm.nih.gov/pmc/journals/378/), ensuring the long-term accessibility of the growing body of knowledge recorded in the journal and by the growing community of researchers from the region. It is encouraging to know that across Africa, the number of journals that are becoming OA is growing, as is awareness about institutional repositories, thanks to the efforts of organizations such as the Electronic Information for Libraries (http://www.eifl.org/) and the Electronic Publishing Trust for Development (http://www.epublishingtrust.org/), the latter of which all three authors are trustees.

## Structural Inequity in Current Reward Systems

Another major potential of OA is the correction to the current structural problem of the academic evaluation and reward system, which has been dominated by a set of narrowly defined citation measures, most notably the journal impact factor (JIF), owned and controlled by the information conglomerate Thomson Reuters. The consolidation of the JIF as a global yardstick for measuring the quality of journals has created a highly competitive landscape of journal ranking and citation gaming, with journals from the developing countries being consistently marginalized [4],[5].

This structural inequality has resulted in a citation and reputation divide in the developing world, with a sub-community of authors who publish almost exclusively in “international” journals indexed in the Thomson Reuters (formerly ISI) Web of Knowledge, while others are oriented towards research and publication in “local” journals on topics of interest to “local” audiences [6]. And even though the latter may have greater impact for local or regional economic growth and public policy, these publications are often neglected by international funders because of the lack of an ISI-recognized citation. This underscores the need to expand the range of metrics or indicators of impact that take into account how “local” scholarship and scientific reporting affect a variety of development impacts and social outcomes.

## Global Knowledge Commons

Acceptance of new forms of metrics for measuring research impact and adoption by the funding agencies would require a substantial cultural shift, but this is a great potential of OA that must be heeded. At the same time, there needs to be a fundamental shift from thinking of knowledge as private property for national competitive advantage, to the collective thinking of knowledge as a Global Public Good [7], much as fresh water and the air that we share. In the highly interconnected world we live in with the constant movement of people and livestock, it is well understood that phenomena such as communicable diseases and climate-related environmental changes do not recognize national boundaries, much less abstract measures such as gross domestic product (GDP). The sharing of knowledge discovery across borders and the building of a global knowledge commons is increasingly important for solving problems that we all face.

But the financing of a global knowledge commons and its governance remains one of the most intractable problems today, because there is no world body that possesses the authority to tax globally in order to finance the production of global public goods [8], and supranational organizations such as the WHO and the Food and Agriculture Organization have no mandate to take on such roles. As a form of “new commons”, the global knowledge commons enabled by OA is still poorly understood because of its infancy, and it requires more concerted study from across disciplines in terms of its governance and sustainability [9].

But there are already important lessons we can learn from the success of OA so far, and from the world of open source software and what Benkler [10] has called non-market commons–based peer production, of which Wikipedia is the best-known example. The power of the network is profoundly transforming the nature of scientific discovery, reporting, and collaboration, and the days of traditional journals must be numbered. Experimentations with new forms of scholarly communication and new forms of metrics abound and researchers are at the forefront of leading the changes. See, for example, the recent paper on “Wikipedia: A Key Tool for Global Public Health Promotion” [11]. See also the recent workshop titled “Beyond the PDF”, and the variety of models, publishing tools, and impact metrics being developed by scientists interested in a more efficient means of collaborating and communicating research results [12].

## The Invisible College

The advent of the Web and the shift from “Big Science” to networked science creates unprecedented opportunities for developing countries to tap OA's potential and contribute on an equal footing. Rather than investing scarce resources in retrograde efforts to mimic or duplicate the scientific institutions and practices of the past century, developing country policymakers can leverage networks by creating incentives for scientists to focus on research that addresses their concerns and by finding ways to mobilize knowledge for local problem solving. As network accessibility across Africa and other developing regions continues to grow, it is important that researchers begin to take full advantage of the new networking tools and collaborative opportunities to address local issues as well as to attain international research opportunities on limited resources. We are all part of what Caroline Wagner called the “New Invisible College”, a global networked college based on mutual interests and open sharing of knowledge, and free from market control of public goods [13]. This highly distributed college is the foundation for the new knowledge commons where the GDP of the country where one resides is neither a passport nor a barrier to participation.

The OA movement, driven as it is by the Invisible College, is an opportunity to re-think not only the equal distribution of all research knowledge, but to reconsider the way in which knowledge is valued and measured.

## Abbreviations

HINARI: Health InterNetwork Access to Research Initiative
JIF: journal impact factor
OA: open access
UN: United Nations
WHO: World Health Organization
